# Exercise and the Timing of Snack Choice: Healthy Snack Choice is Reduced in the Post-Exercise State

**DOI:** 10.3390/nu10121941

**Published:** 2018-12-07

**Authors:** Christopher R. Gustafson, Nigina Rakhmatullaeva, Safiya E. Beckford, Ajai Ammachathram, Alexander Cristobal, Karsten Koehler

**Affiliations:** 1 Department of Agricultural Economics, University of Nebraska-Lincoln, Lincoln, NE 68583, USA; cgustafson6@unl.edu (C.R.G.); n.rakhmatullaeva@gmail.com (N.R.); 2 Department of Nutrition and Health Sciences, University of Nebraska-Lincoln, Lincoln, NE 68583, USA; safiya.beckford@huskers.unl.edu (S.E.B.); ajai@unl.edu (A.A.); acristobal0474@huskers.unl.edu (A.C.)

**Keywords:** compensatory eating, exercise-induced anorexia, food choice, acute exercise, behavioral economics, nudges

## Abstract

Acute exercise can induce either a compensatory increase in food intake or a reduction in food intake, which results from appetite suppression in the post-exercise state. The timing of food choice—choosing for immediate or later consumption—has been found to influence the healthfulness of foods consumed. To examine both of these effects, we tested in our study whether the timing of food choice interacts with exposure to exercise to impact food choices such that choices would differ when made prior to or following an exercise bout. Visitors to a university recreational center were equipped with an accelerometer prior to their habitual workout regime, masking the true study purpose. As a reward, participants were presented with a snack for consumption after workout completion. Participants made their snack choice from either an apple or chocolate brownie after being pseudo-randomly assigned to choose prior to (“before”) or following workout completion (“after”). Complete data were available for 256 participants (54.7% male, 22.1 ± 3.1 years, 24.7 ± 3.7 kg/m^2^) who exercised 65.3 ± 22.5 min/session. When compared with “before,” the choice of an apple decreased (73.7% vs. 54.6%) and the choices of brownie (13.9% vs. 20.2%) or no snack (12.4% vs. 25.2%) increased in the “after” condition (χ^2^ = 26.578, *p* < 0.001). Our results provide support for both compensatory eating and exercise-induced anorexia. More importantly, our findings suggest that the choice of food for post-exercise consumption can be altered through a simple behavioral intervention.

## 1. Introduction

Regular exercise and a healthy diet are important staples of a healthy lifestyle. The beneficial effects of exercise for the treatment and prevention of many physiological and psychological conditions, including diabetes, cardiovascular disease, certain cancers, recovery from stroke, emotional well-being, depression, anxiety, and suicidal behaviors [1,2,3,4,5,6,7,8], are well documented. However, the impact of exercise on overweight and obesity, and particularly its ability to produce meaningful weight loss remains under debate [9]. It is undisputed that exercise increases energy expenditure and thereby has the potential to induce weight loss. However, individual weight loss responses are mixed [10], suggesting that the success of exercise as a weight loss strategy is largely dependent on its effects on the other component of energy balance, i.e., dietary energy intake. A primary barrier to exercise-induced weight loss is compensatory eating, which is defined as an increase in food intake following exercise or physical activity [11].

It is a common belief that exercise stimulates appetite and food intake [12], and as much as 77% of college students report engaging in compensatory eating [11,13]. The mechanisms for this compensatory increase in food intake following exercise are manifold, but most likely include endocrine pathways that favor food intake to ensure the maintenance of body weight, and more specifically lean body mass [14,15]. On the other hand, there is also evidence in the literature contrary to compensation. Evidence shows that only 19% of intervention studies reported an increase in energy intake after exercise, whereas 65% show no change [16]. Furthermore, it has repeatedly been demonstrated that appetite and hunger are suppressed following exercise, particularly in the immediate post-exercise state [12]. This reduction in perceived hunger has been termed “exercise-induced anorexia” and has been linked to the suppression of orexigenic hormones, such as ghrelin, and concomitant increases in satiety hormones, including peptide YY and glucagon-like peptide 1 [17,18,19].

While the impact of exercise on energy intake is important from an energy balance perspective, it is critical to understand that energy intake is ultimately the product of food choices, as individuals select foods rather than nutrients under free-living conditions [12]. It has been proposed that exercise participation can impact food selection, modify the sensitivity to sensory cues [12], and alter the reward value of foods with particular sensory and/or macronutrient profiles [20,21]. Hedonic mechanisms controlling food intake are stimulated by the sensory pleasure of eating palatable food and may result in increased food intake [22]. This increase in food intake has been linked to the neuroendocrine factor dopamine, which has been shown to reinforce the pleasure derived from eating highly palatable foods [23,24]. Exercise has been found to reduce addictive behaviors such as drug and alcohol consumption [23], and as such it is possible that exercise may also elicit a reduction in food intake as a result of the rewarding value of food. Previous experiments have shown that the responsiveness of brain regions related to food reward is altered in the post-exercise state [25], such that the brain’s neural reward system’s response to low energy density foods is increased and the reaction to high energy density is reduced [26,27,28].

In support of the contradictory findings in the literature, it has been proposed that exercise could either increase or decrease the reinforcing value of energy-dense, palatable foods. For example, the deliberate choice of highly palatable, energy-dense foods (e.g., fatty and/or sweet “treats”) in the post-exercise state has been linked to compensatory eating and reduced weight loss success [21]. Others have reported that carbohydrate-rich foods are rated more palatable in the post-exercise state [25,26], possibly reflecting increased carbohydrate utilization during aerobic exercise [29]. Alternatively, exercise could also reduce the consumption of these foods as a result of improved appetite control coupled with a higher motivation to engage in healthy behaviors [28]. These diametrically opposed effects of exercise on food choices could ultimately explain the inter-individual variation in the degree of compensatory eating following exercise interventions [21].

Another important influence on dietary quality is the timing of food choice relative to consumption. Prior research from behavioral economics and psychology suggests that changing the timing of food choice relative to consumption—whether food choices will be consumed immediately or at some later point—influences the healthfulness of the foods chosen [30,31]. Behavioral economic models of choices over time (the most prominent of which is hyperbolic discounting [32]) have been formulated to represent individuals who have inconsistent preferences. These models predict that choices will differ depending on whether the chosen item is to be received immediately or after a delay. When the decision-maker will receive their choice immediately, the models predict that the individual will make less healthy, more impatient decisions than if the receipt is delayed. Individuals whose choices fit this pattern are said to have “present-biased preferences.” An upshot of present-biased preferences is that people will often be willing to pre-commit to a healthier behavior if given the opportunity to do so [33,34].

To integrate the impact of biophysical and behavioral effects on post-exercise food intake in a real-life scenario, the overall goal of the present study was to test whether manipulating the timing of the choice of a snack to be received after exposure to exercise would alter food choices such that in the post-exercise state the choice of snacks with varying energy density and health attributes would differ from choices made prior to exercise. Our intervention addresses a simple but important question: *Can a simple nudge—changing the time when the choice of a post-exercise snack is made—help individuals select healthier food options?* Given the importance of understanding why people make unhealthy dietary choices and how these choices can be discouraged [35], examining how factors such as the timing of food choice that may promote healthy eating in the context of exercising would be beneficial [28]. This is particularly true since behavioral weight loss strategies typically employ a combination of diet and/or exercise. However, as mentioned previously, evidence suggests that increases in food consumption in response to exercise can derail weight loss efforts [16]. Knowledge about the degree to which food choices are altered from pre- to post-exercise could ultimately allow individuals to pre-commit to healthier food choices by selecting food in a state which favors their choice of healthier options. Our primary hypothesis was that individuals are more likely to choose an “unhealthy,” energy-dense snack in the post-exercise state when compared to choosing a snack for post-exercise consumption prior to exercising, supporting previous evidence of compensatory eating [11] as well as findings from behavioral studies of food choice [30]. To also account for previously reported reductions in appetite and hunger in the immediate post-exercise state, we further hypothesized that the number of participants who would decline a snack would also increase in the post-exercise state.

## 2. Materials and Methods

### 2.1. Study Design

The experiment was conducted at the University of Nebraska-Lincoln Recreational and Wellness Center and was focused on the effect of the timing of food choice—which was assigned by the researchers to take place before or after the participant exercised—on selection of a food item. The food item was received after the exercise was completed in both conditions. The experiment took place on randomly chosen weekdays between February 16 and April 30, 2018, and implementation of conditions was balanced across the study period and days of the week. Individuals were recruited to participate as they entered the recreational center. All participants had to be at least 19 years of age and to have come to the Recreational and Wellness Center to exercise. When individuals were invited to participate, the purpose of the study was presented as being focused on calibrating activity sensors to various exercise types. As an incentive for participation in this “calibration study,” participants were offered a choice of food items upon completion of the study. In actuality, participants’ choice of a food item was of central interest to the study, but this interest in participants’ food choices was not highlighted to minimize experimenter demand effects [36] and social desirability biases [37,38]. Participants signed a written informed consent form prior to the experiment. The study was approved by the University of Nebraska-Lincoln Institutional Review Board.

### 2.2. Procedures

After participants signed the informed consent form, study staff measured their height and weight on a digital column scale (Seca, Hamburg, Germany). Next, participants were fitted with an accelerometer (GT3X, ActiGraph, Pensacola, FL, USA) on their non-dominant wrist, which they wore for the duration of their workout. Study staff instructed all participants to proceed with the workout that the participant had planned to complete at the Recreation and Wellness Center prior to study recruitment.

When subjects returned the accelerometers upon completion of their workout, additional information was collected, including each participant’s date of birth, the type of activity or activities conducted during the workout, and whether participants ate or drank anything during the workout. Participants were told that this information was needed to personalize the accelerometer data.

### 2.3. Food Choice Paradigm

In order to examine changes in food choices over the course of a self-selected workout, participants were given a snack choice to be received upon completion of their workout as a reward for study participation. In one condition, prior to beginning their workout (“before”), participants were asked to choose which snack item they wanted to receive. Upon completion of their workout and them returning the accelerometer, participants were given the food item they had selected. In the other condition (“after”), participants were asked to choose their snack right as they came back to return their accelerometer after their workout. In the “after” condition, participants received their snack immediately after making their choice. For logistical purposes, one condition (“before” or “after”) was implemented per study day.

In order to present two snack options with distinguishable perceived health attributes but similar taste attributes (sweet), participants were able to choose between an apple (deemed “healthy”) and a brownie (deemed “unhealthy”). Participants also had the option to decline both snacks (“neither”). Both snack options were visible to participants as they checked in (“before” condition) or as they checked out (“after” condition). Prior to giving the chosen snack to the participant, study staff inquired about food allergies and intolerances.

All food used in the experiment was purchased and prepared in a large batch by a food and beverage management specialist in a department certified kitchen and stored at appropriate conditions (temperature checks were performed twice per day) prior to presenting it to study participants. The apple variety offered in the study was Fuji with an average energy content of 121 kcal per medium-sized apple [39]. Brownies were prepared from a prepackaged commercial brownie mix (Ghiradelli, San Leandro, CA, USA) and had an energy content of 140 kcal per piece.

### 2.4. Data Analyses

Data were analyzed using R Statistical Software (R Core Team, R Foundation, Vienna, Austria). Prior to data analysis, data from individuals who participated in the study multiple times were eliminated from the dataset such that only the first study visit was included in the analyses. In addition, data from participants who reported food allergies or intolerances that could have affected the food choice were eliminated from the dataset. Participants’ food item choices were analyzed to evaluate whether the proportion of choices of the different food items (including “neither”) differed by condition using a chi-squared test. We then used multinomial, multivariate logistic regression models to examine the relationship between food choice and condition while controlling for potentially confounding variables comprising body mass index (BMI) category (≤25 kg/m^2^; >25 kg/m^2^), age (in years), gender, workout duration (in minutes), whether the participant consumed any food during their workout, and mode of exercise (aerobic, resistance, and other). BMI categories were aggregated from four initial categories (underweight, normal weight, overweight, and obese) into two (underweight/normal weight, and overweight/obese) because few participants fell into the underweight (3 participants) and obese (18 participants) categories. Results of the multinomial logistic regression models are presented as odds ratios (OR) and 95-percent confidence intervals (95% CI) for independent variables. Statistical significance was considered for *p*-values < 0.05.

## 3. Results

A total of 299 data points were initially collected. Data from 31 participants (42 observations) were eliminated because individuals had participated in the study more than once. In addition, data from one participant who reported having celiac disease were omitted. The final dataset used for analysis contained observations from 256 unique participants. Of these, 137 participants (53.5%) completed the “before” condition and 119 completed the “after” condition (46.5%). On average, 54.7% of participants were male, 22.1 ± 3.1 years old, had an average BMI of 24.7 ± 3.7 kg/m^2^, and exercised for 65.3 ± 22.5 min. There were no significant differences between conditions in gender distribution, age, BMI or BMI categories, workout duration, food consumption, or mode of exercise (Table 1).

**Figure 1:**
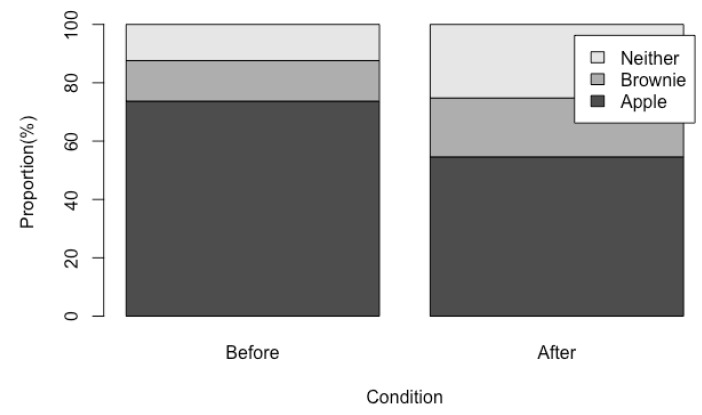
Proportion of snack choices (apple, brownie, or neither) for consumption after completion of a workout chosen either before the beginning of the workout (“before” condition) or after completion of the workout (“after” condition).

In the “before” condition, 101 participants (73.7%) selected an apple, 19 (13.9%) selected a brownie, and 17 (12.4%) declined a snack upon completion of their workout. In the “after” condition, 65 participants (54.6%) selected an apple, which is an ~20% decrease from the “before” condition. Twenty-four participants (20.2%) selected a brownie in the “after” condition, and 30 participants (25.2%) declined a snack option upon completion of their workout. The patterns of choices (Figure 1) differed significantly between the “before” and “after” condition (χ-squared = 26.578, df = 2; *p* < 0.001).

Results from the multinomial, multivariate logistic regression model are reported in Table 2. The omitted snack choice was “neither” in the regression model. Results confirm that the odds of a participant choosing an apple decreased significantly in the “after” condition relative to the choice of neither (OR: 0.33; 95% CI: 0.16–0.66), even after controlling for other variables. The estimated odds ratio for the effect of the “after” condition on apple choice remained unchanged regardless of other independent variables included in the regression model, including BMI status, age, gender, and workout duration. There were no significant differences in brownie choice (relative to choosing neither) based on condition (OR: 0.64; 95% CI: 0.26–1.55). In addition to the condition, only the BMI category significantly contributed to the regression model of snack choice. Individuals who were classified as overweight or obese (BMI > 25 kg/m^2^) were significantly less likely to select a brownie than individuals with a BMI < 25 kg/m^2^ (OR: 0.37; 95% CI: 0.15–0.92).

## 4. Discussion

The goal of the present study was to assess whether the timing of snack choice would interact with exposure to a single exercise bout to alter food choices. Using a behavioral intervention approach, we show that a very simple modification—making a choice about a post-exercise snack either prior to or following the completion of the exercise bout—significantly alters the snack choice. Our findings indicate that the likelihood of choosing an apple, a food typically considered as “healthy”, is about one third (33.5%) greater when the choice is presented prior to engaging in exercise; however, when the choice is presented following the exercise bout, individuals are approximately 39% more likely to choose a brownie, a food typically considered as “unhealthy”, and 112% more likely to decline either snack option. These findings exhibit elements of two previously identified effects of exercise on food choice: compensatory eating, which refers to the increase in food intake following exercise of physical activity [11], and exercise-induced anorexia, which refers to a temporary reduction in appetite immediately following exercise [40]. They also correspond to patterns seen in previous behavioral research, in which individuals are more likely to make healthier choices if the food will be delivered in the future rather than immediately [30].

### 4.1. Interindividual Variation in Post-Exercise Food Choices: Compensatory Eating and Exercise-Induced Anorexia

The increase in the preference for a brownie, an energy-dense and palatable snack, can be seen as evidence for compensatory eating. Finlayson et al. speculated that hedonic processes are modulated by increased energy expended during exercise, thereby promoting overconsumption in individuals prone to compensatory eating [22]. Considering that a greater tendency for compensatory eating has been linked to attenuated weight loss during exercise interventions [21], our findings highlight the importance of timing of food choices for individuals who exercise to lose weight. In contrast, others have reported a reduced preference for energy-dense food following exercise. For example, an acute bout of resistance exercise decreased the preference for high-fat food [27], and 2 weeks of aerobic exercise were found to reduce the reinforcing value of high energy density foods [28]. However, it is noteworthy that there was a dose-dependent relationship between habitual exercise and the reduction in the reinforcing value of high energy density foods, which was most pronounced in individuals who exercised 5 days per week when compared to individuals who exercised less regularly [28].

In addition to an increased preference for an “unhealthy” option, we further observed an increase in the number of individuals who declined either food option among participants who were given a snack choice in the post-exercise state. While we acknowledge that there may be other reasons for this finding, this finding is in accordance with previous reports of exercise-induced anorexia. This phenomenon, which describes a transient reduction in appetite and hunger in the immediate post-exercise state, has been linked to reductions in ghrelin, an appetite-stimulating hormone, and increased concentrations of satiety hormones, including peptide YY and glucagon-like peptide 1 [17,18,19,41]. However, hunger is not necessarily indicative of actual food intake, as shown in a study involving exposure to exercise message commercials, which resulted in higher ratings of hunger but lower caloric intake [42].

Taken together, increases in compensatory eating and exercise-induced anorexia result in a greater inter-individual variability in post-exercise food intake. These competing effects, i.e., an increase in consumption of an “unhealthy” option along with a greater number of individuals declining either food option, negate an overall group effect. In fact, when determining the caloric intake based on food choices made (brownie, apple or neither), average caloric intake did not change dramatically from “before” (109 kcal/participant) to “after” (94 kcal/participant), which is in support of a previous meta-analysis by Schubert et al. who failed to identify a definite effect of an exercise task on post-exercise caloric intake [10]. This finding is also in agreement with observations from our laboratory, according to which inter-individual differences in food choices are greater in the post-exercise state when compared to the rested state (Koehler, unpublished observation).

### 4.2. Impact of Gender and Body Mass Index

In contrast to previous studies, we failed to observe a significant gender effect in our food choice paradigm. Although most laboratory experiments addressing the impact of a single exercise bout on food intake were not specifically designed to detect gender differences, it is well established in the literature that food intake patterns differ between men and women and that women consistently make healthier choices than men [43]. Furthermore, the motivation to exercise tends to differ between genders, as women are more likely to exercise in attempts to lose weight [44]. As such, it is not surprising that when compared to their male counterparts, female athletes exhibit more self-control for high-calorie and sweet food options [45]. However, despite these gender differences, a recent meta-regression showed no impact of gender on post-exercise calorie intake [10], which is in agreement with the lack of a gender effect in our study. This lack of gender difference may be further explained by the fact that a greater proportion of our female participants conducted aerobic exercise (84% vs. 52% in males, *p* < 0.001), which is reflective of the general exercise motives of young adults [46]. It is possible that the lower energy expenditure of resistance exercise, when compared to aerobic exercise [47], reduced the likelihood of compensatory eating in the male participants. Furthermore, a recent study reported that resistance exercise, but not aerobic exercise, reduced explicit liking for high-fat food [27].

We did, however, find an effect of BMI on overall food choice, whereby individuals with a BMI indicative of overweight or obesity (> 25 kg/m^2^) were less likely to choose our “unhealthy” option. This finding is contrary to previous literature suggesting that obesity is associated with an increased drive to eat palatable foods [48], specifically in the absence of hunger [49], and greater impulsivity toward food reward [50]. However, it should be noted that the current study was selectively conducted in individuals who voluntarily exercised, a group not necessarily representative of individuals who are overweight and obese [51]. As such, our overweight or obese participants may have exhibited a greater motivation to lose weight compared to non-exercising individuals [52]. Furthermore, it is also possible that our measure of BMI overestimated levels of overweight and obesity in our group of exercisers, as it is well known that BMI fails to differentiate between individuals with excess adiposity vs. individuals with increased muscle mass [53].

### 4.3. Limitations

As the study presents a simple behavioral manipulation in a real-life setting, there are various limitations to our investigation. First, we failed to assess changes in ratings of appetite or hunger over the course of the exercise bout as well as appetite-regulating peptides, and more specifically whether inter-individual differences in these outcomes would explain differences in food choices from pre- to post-exercise. However, as previously mentioned, it is well known that hunger levels alone fail to explain differences in the food intake response following exercise [22].

In order to test the impact of exercise on food choice in a real-life scenario, we chose to allow participants to follow their regular exercise regimen. As such, exercise intensity was self-selected and consequently varied among our participants. High-intensity exercise has been shown to favor a negative energy balance to a greater extent than low-intensity exercise [54]. Although each participant wore an accelerometer during their exercise bout, we chose not to include this data in the present analysis due to previous studies demonstrating that the Actigraph tends to perform poorly during vigorous activities [55], which we presumed to be the primary intensity range in the present study. Regardless, future studies should attempt to assess exercise intensity using objective (e.g., heart rate) or subjective (e.g., ratings of perceived exertion) measures. Another limitation was that we did not assess the prandial state, such as the time of the last meal or current hunger levels. While intriguing, these measures were not taken in order to avoid revealing the true purpose of this investigation. We did, however, assess food intake (in excess of water) during the workouts, but this variable did not have a significant effect on food choice as shown by our regression analysis (Table 2). While future studies should more carefully monitor food intake and hunger levels prior to the experiment, we are confident that using a relatively large sample and conducting all experimental trials at the same time of day minimized the impact of the prandial state on our outcomes.

Our findings clearly highlight that food choices change depending on whether a post-exercise snack is chosen prior to or following the exercise bout. Behavioral economic models of intertemporal choice provide another perspective on this pattern of choice [32]. Many people have inconsistent preferences for outcomes that occur at different time points relative to when the choice is made. While most of the evidence is for choices made over monetary outcomes, there is some evidence about food choice as well. When people make choices for foods that they will receive immediately, time-inconsistent preferences tend to lead them to be more indulgent—choosing less healthy foods—than if receipt of the selected food is in the future. This model predicts that people are more likely to make a healthy choice for their future self than they are for their current self [56]. While this phenomenon may have explained the increased preference for the “unhealthy” option (brownie) following the workout (immediately before the snack) compared to before the workout (on average 65 min before their snack), it fails to explain the increase in the number of participants who declined either snack. A second behavioral economic influence may shed light on increases in both unhealthy and neither food option: projection bias [57]. Projection bias refers to the failure of a decision-maker to correctly predict their preferences for outcomes that occur in the future. This bias is thought to be particularly likely to occur when the individual is making a choice in one state that will be experienced in another state [58]. It is well established that exercise induces changes in an individual’s state by, for instance, suppressing appetite-inducing hormones [17,18,19,41]. Future studies can feature more sophisticated designs that separately identify these various influences on decision-making, such as including non-exercising control activities to disentangle the effects of time-inconsistent preferences from compensatory eating and exercise-induced anorexia and varying the state in which participants make food choices for immediate and future receipt.

## 5. Conclusions

Overall, our study demonstrates that the choice of a post-exercise snack can be shifted through a simple behavioral intervention, i.e., choosing the snack prior to or after the exercise bout. As such, our results provide support for both an increased preference for compensatory eating as well as an increased degree of exercise-induced anorexia. Our findings have important practical implications for individuals who are attempting to lose or control their weight through exercise, as well as for health professionals providing guidance and support to these individuals. Corroborating previous research [34,58], participants in this study who chose their snack before they exercised—the “before” condition—were more likely to select a healthier snack than participants who chose immediately prior to receipt of the snack (the “after” condition). A simple strategy such as encouraging individuals to make choices about foods that they will eat post-exercise prior to their workout may help those who are attempting to lose weight through diet and exercise pre-commit to healthier foods. Pre-commitment can prevent individuals from offsetting the gains in exercise-related caloric expenditure through compensatory eating.

## Figures and Tables

**Table 1:** Characteristics of the study sample.

	All Participants (*n* = 256)	“Before” Condition (*n* = 137)	“After” Condition (*n* = 119)	*p*-Value *
Age	22.1 ± 3.1	22.0 ± 2.9	22.1 ± 3.4	0.72
Gender (Male)	135 (52.7%)	75 (54.7%)	60 (50.4%)	0.49
BMI (kg/m^2^) Underweight/Normal weight Overweight/Obese	24.7 ± 3.7 148 (57.8%) 108 (42.2%)	24.8 ± 3.6 75 (54.8%) 62 (45.2%)	24.6 ± 3.8 73 (62.3%) 46 (38.7%)	0.55
				0.29
				0.29
Workout Duration (min)	65.3 ± 22.5	67.3 ± 25.5	63.0 ± 18.3	0.12
Food Consumption (Yes)	8 (3.1%)	5 (3.6%)	3 (2.5%)	0.60
Aerobic Exercise (Yes)	172 (67.2%)	87 (63.5%)	85 (73.4%)	0.18
Resistance Exercise (Yes)	192 (75%)	102 (74.5%)	90 (75.6%)	0.83
Other Exercise (Yes)	9 (3.5%)	7 (5.1%)	2 (1.7%)	0.13

* “before” vs. “after”.

**Table 2:** Odds ratios (OR) and 95% confidence intervals (CI) from multinomial logistic regression results of snack choice, relative to neither.

	Apple		Brownie	
	OR	95% CI	OR	95% CI
Condition: “After”	0.33	(0.16–0.66)	0.64	(0.26–1.55)
BMI Status (>25 kg/m^2^)	0.61	(0.31–1.23)	0.37	(0.15–0.92)
Age	0.98	(0.87–1.10)	1.09	(0.96–1.24)
Gender (male)	1.17	(0.56–2.43)	1.81	(0.71–4.64)
Workout Duration (min)	1.00	(0.98–1.02)	1.00	(0.98–1.02)
Food Consumption (Yes)	1.51	(0.17–13.62)	1.16	(0.07–20.00)
Aerobic Exercise (Yes)	1.11	(0.48–2.59)	0.85	(0.30–2.40)
Resistance Exercise (Yes)	1.03	(0.43–2.43)	0.91	(0.30–2.77)
Other Exercise (Yes)	0.47	(0.04–1.56)	0.47	(0.05–4.45)
Intercept	10.65	(0.53–214.60)	0.31	(0.01–9.75)

Number of observations = 256, Akaike Information Criterion = 470.9.
